# Emerging Roles of Non-proteolytic Ubiquitination in Tumorigenesis

**DOI:** 10.3389/fcell.2022.944460

**Published:** 2022-07-06

**Authors:** Xiu Yin, Qingbin Liu, Fen Liu, Xinchen Tian, Tinghao Yan, Jie Han, Shulong Jiang

**Affiliations:** ^1^ Clinical Medical Laboratory Center, Jining First People’s Hospital, Jining Medical University, Jining, China; ^2^ Cheeloo College of Medicine, Shandong University, Jinan, China; ^3^ Department of Thyroid and Breast Surgery, Jining First People’s Hospital, Jining Medical University, Jining, China

**Keywords:** ubiquitin, atypical ubiquitination, ubiquitin E2 conjugating enzyme, ubiquitin E3 ligase, tumorigenesis, ubiquitin-proteasome system

## Abstract

Ubiquitination is a critical type of protein post-translational modification playing an essential role in many cellular processes. To date, more than eight types of ubiquitination exist, all of which are involved in distinct cellular processes based on their structural differences. Studies have indicated that activation of the ubiquitination pathway is tightly connected with inflammation-related diseases as well as cancer, especially in the non-proteolytic canonical pathway, highlighting the vital roles of ubiquitination in metabolic programming. Studies relating degradable ubiquitination through lys48 or lys11-linked pathways to cellular signaling have been well-characterized. However, emerging evidence shows that non-degradable ubiquitination (linked to lys6, lys27, lys29, lys33, lys63, and Met1) remains to be defined. In this review, we summarize the non-proteolytic ubiquitination involved in tumorigenesis and related signaling pathways, with the aim of providing a reference for future exploration of ubiquitination and the potential targets for cancer therapies.

## 1 Introduction

### 1.1 The Ubiquitin-Proteasome System

Ubiquitination, also known as ubiquitylation, refers to the process by which ubiquitin (Ub, a small and highly conserved protein), with the help of a series of special enzymes, classifies proteins in cells, selects target proteins, and modifies those proteins (McDowell and Philpott, 2016; Seeler and Dejean, 2017; Rape, 2018). Ubiquitination plays fundamental roles in many cellular events such as cell proliferation (Kwon and Ciechanover, 2017; Werner et al., 2017; Senft et al., 2018; Song and Luo, 2019), cell cycle (Teixeira and Reed, 2013; Darling et al., 2017; Gilberto and Peter, 2017), DNA repair (Alpi and Patel, 2009; Abbas and Dutta, 2011), immune response (Heaton et al., 2016; Rudnicka and Yamauchi, 2016; Manthiram et al., 2017), transcription (Zhou et al., 2018; Imam et al., 2019; Sun et al., 2019), angiogenesis (Zhang et al., 2022), metastasis (Rossi and Rossi, 2022), and apoptosis (Xu et al., 2017; Zhou et al., 2017).

**FIGURE 1:**
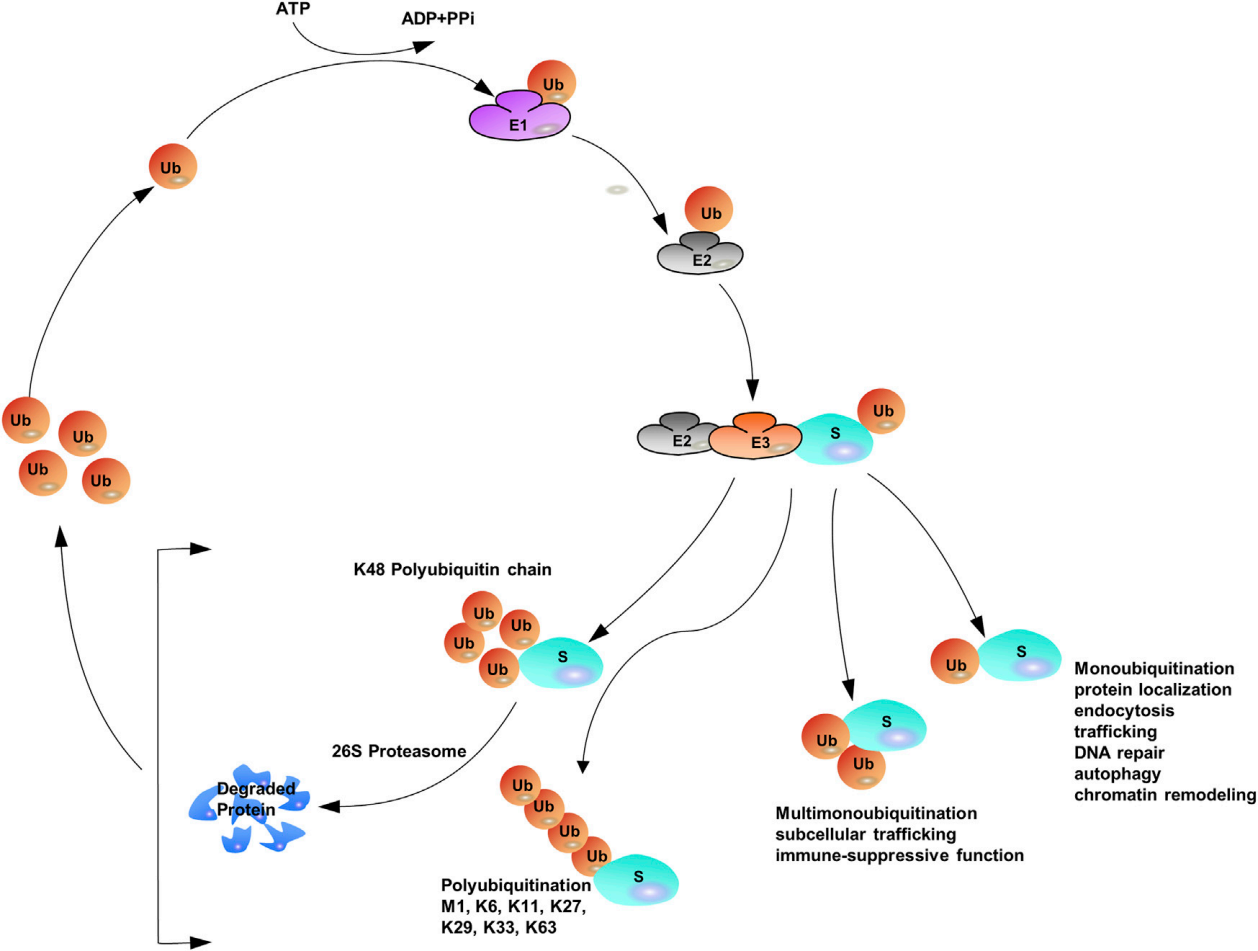
The Ubiquitination Machinery: ubiquitin conjugates can differ in structure and function.

Protein ubiquitination requires three different enzymes: E1 ubiquitin-activating enzymes, E2 ubiquitin-conjugating enzymes, and E3 ubiquitin ligases (Heap et al., 2017; O'Connor and Huibregtse, 2017). E1 enzymes activate the ubiquitin polypeptide in an ATP-dependent manner, and the activated forms are then conjugated to E2 enzymes through the formation of thioester bonds (Wang et al., 2018). Finally, E3 ubiquitin ligases recognize both E2 enzymes and specific target substrates that confer specificity to the system, and then the Ub can be transferred from E2 enzymes to the target substrates to complete the ubiquitination process (Rittinger and Ikeda, 2017; Yau et al., 2017). The coordination of E1, E2, and E3 enzymes earmarks target proteins with a wide variety of ubiquitin modifications such that distinct ubiquitin modifications transmit different cellular signals (Khaminets et al., 2016; Venuto and Merla, 2019). Specific ubiquitin-binding domains (UBDs) can identify ubiquitylated substrate proteins (Kristariyanto et al., 2015; Kung et al., 2019; Polykratis et al., 2019; Spiliotopoulos et al., 2019). UBDs utilize diverse mechanisms to interact with various surface patches on ubiquitin molecules or different ubiquitin linkages (Hicke et al., 2005; Dikic et al., 2009; Komander and Rape, 2012). The hydrophobic surfaces of ubiquitin molecules, such as isoleucine 36 (Ile36) and isoleucine 44 (Ile44), are the structural basis for the recognition of ubiquitination signals. Different ubiquitin chains have various spatial structures and thus expose different hydrophobic surfaces, which can be recognized by specific UBDs. Proteins containing UBDs recognize and transmit the functional signals represented by ubiquitin chains (Buetow and Huang, 2016). Similar to many other protein post-translational modifications (PTMs), ubiquitination can be preserved through cleavage by deubiquitylating enzymes (DUBs) (Herhaus and Sapkota, 2014; Nishi et al., 2014; Clague et al., 2019) (Figure 1).

The versatility of ubiquitination is determined by the complex assembly pattern of ubiquitin molecules on the target protein. Ubiquitins can be attached to one or multiple lysine residues with either a single ubiquitin molecule (mono- and multi-mono-ubiquitination, respectively) or ubiquitin polymers (poly-ubiquitination) (Eger et al., 2010; Akutsu et al., 2016; Chitale and Richly, 2017; van der Heden van Noort et al., 2017). Poly-ubiquitin chains comprising only one single linkage are often assumed to be homotypic (Jeusset and McManus, 2017; Leestemaker and Ovaa, 2017), whereas heterotypic chains adopt multiple linkages within the same polymer (branched or non-branched) (Akturk et al., 2018). In a poly-ubiquitin chain, ubiquitin moieties can be linked through any of the seven lysine residues (K6, K11, K27, K29, K33, K48, and K63) or N-terminal methionine (Met1) (Dittmar and Winklhofer, 2019; Spit et al., 2019; Liao et al., 2022), resulting in an almost unlimited number of poly-ubiquitin chain topologies (Varadan et al., 2004; Sadowski and Sarcevic, 2010; Dondelinger et al., 2016; O'Connor and Huibregtse, 2017; Padala et al., 2017). Further complexity is added to ubiquitination when the ubiquitin polypeptide is modified by phosphorylation (Dong et al., 2017) or acetylation (Choudhary et al., 2009; Ohtake et al., 2015). Given the sophisticated assembly of protein ubiquitination, it has been often referred to as the “ubiquitin code” (Komander and Rape, 2012). Proteomics studies have shown that Lys48-linked chains are predominant in cells (>50% of all linkages), and Lys63-linked chains rank the second abundant chain form, however, researchers have begun to characterize the remain chain types, which were considered to be “atypical” ubiquitin modifications (linked through Lys6, Lys11, Lys27, Lys29, Lys33 and Met1) (Xu et al., 2009; Dammer et al., 2011; Kim et al., 2011; Wagner et al., 2011; Ziv et al., 2011).

### 1.2 Structural Features of Poly-Ubiquitin Chains

Poly-ubiquitin chains occur when a single ubiquitin molecule is repeatedly connected in series with another ubiquitin lysine residue. Substrate proteins can be distinguished by poly-ubiquitin chains by attaching between different types of deubiquition formations, single mono-ubiquitination events, multiple mono-ubiquitinations events, homotypic ubiquitination events and heterotypic ubiquitination events (branched and non-branched ubiquitination) (Sokratous et al., 2014; Yau and Rape, 2016; Zhao et al., 2017). This also led to the formation of eight different homotypic chains. The key distinguishing feature of how this can be achieved is the specific combination of E2/E3 enzymes, thereby triggering distinct cellular fates of substrate proteins. However, some of the reported E2/E3 enzyme combinations were not 100% specific for targeted linkage. It has been reported that two E1 enzymes were selected for ubiquitin in humans: UBA1 and UBA6 (Barghout and Schimmer, 2021). Humans also encoded 40 E2 conjugation enzymes cooperate with approximately 600 E3 ligase enzymes (Hodson et al., 2014). The E3s were categorized into three groups: RING/U-box, HECT, and RING between RING (RBR) (Wang et al., 2020).

With the assistance of E1, E2, and E3, mono-ubiquitination occurs when a single ubiquitin is attached to its target proteins, then Ub molecules are added to the model in linear ways one by one (Torres et al., 2009; Tang et al., 2011). The sequential addition model of Ub on the substrate contributes to the elongation of the Ub lines. When secondary Ub molecules are connected to specific lysine residues, they are called homotypic chains. If any of the attached adjacent Ub molecules are linked to each other by different lysine residues (mixed or branched model), a heteropic structure is formed. For homotypic chains, reports have found that different chain types are closely related to the confirmation of the structure, either “compact” or “open”. Generally, non-proteolytic ubiquitination Lys63 linkages and Met1 linkages, adopt “open” ones. Contrast to the aforementioned linkages, internal structural molecules interact with each other among degradable linkages, like Lys6, Lys11 and Lys48 linkages, and those linkages display “open” conformation. Furthermore, all ubiquitin moieties can be modified by acetylation or phosphorylation to add additional layers of complexity (Kane et al., 2014; Kazlauskaite et al., 2014; Koyano et al., 2014; Ordureau et al., 2014; Ohtake et al., 2015; Swaney et al., 2015) (Figure 1).

Numerous studies have found that ubiquitin acetylation inhibits poly-ubiquitination elongation, and phosphoubiquitin leads to mitophagy. Any PTM chain can be changed to ubiquitin chains, which may prevent or facilitate ubiquitin interactions. Protein phosphorylation is linked to ubiquitination for proteasomal degradation. Reports have shown that the phosphorylation of ULK1 by MAPK1/3 kinase interacts with BTRC, which leads to subsequent proteasomal degradation and attenuates breast cancer bone metastasis (Deng et al., 2020). However, the stability of some proteins is also regulated by phosphorylation. Reports have also shown that Aurora B-mediated phosphorylation of ubiquitin specific protease 13 (USP13) at Serine 114 promoted the stability of Aurora B.

### 1.3 Encoding and Decoding the Ubiquitin Code

Encoding and decoding the ubiquitin code is performed by factors that recognize Ub chains and connect the substrate proteins to the downstream response (Ji and Kwon, 2017; Kwon and Ciechanover, 2017). Recognition of chains occurs through discrete domains and affinity binders with specificity for a particular Ub substrate and chain type (Fu et al., 2012; Suryadinata et al., 2014; Kniss et al., 2018; Michel et al., 2018). This complex system consists of the conjugation of diverse mono, multi-mono and polymeric chains (Rösner et al., 2015; Ji and Kwon, 2017). The interpretation of how, when, and why the ubiquitin codes are written, read, and erased emerged to be characterized. Ubiquitination is a powerful decoration process of proteins and is typically actualized by “ubiquitinase” (Zientara-Rytter and Subramani, 2019). Ubiquitin can be successfully linked to one of the seven lysine residues, all of which can be characterized as poly-ubiquitin chains (Laplantine et al., 2009; Regev et al., 2015; Bax et al., 2019). Poly-ubiquitin chains with different topologies depended upon the lysine residues (which were chosen to be attached) and the substantial chain length, determine the lucky chance of the target proteins and regulate diverse cellular processes, known as “ubiquitin code” (Dittmar and Selbach, 2017; Chatr-Aryamontri et al., 2018; Fottner et al., 2019; O'Donnell, 2019; Song and Luo, 2019). Recent discoveries have deepened our understanding of the whole picture of the ubiquitin code, the interplay between “writers” (E1/E2/E3s), “erasers” (DUBs) and “readers” (ubiquitin binding domain containing proteins) (Tanno and Komada, 2013; Heride et al., 2014; Rogerson et al., 2015; Di Lello and Hymowitz, 2016; Smeenk and Mailand, 2016). Studies have highlighted that mono-ubiquitination can be catalyzed by different E2 and E3 enzymes, acting either individually or together to determine specific substrates. Notably, the linkage specificity of E3s containing RING or U-box domains is likely dictated by E2. As for the HECT E3s class of enzymes, HECT domain swaps can activate the acceptor lysine and are sufficient to determine the linkage specificity. RBR E3s, however, are somewhat complex. RBR E3s display linkage specificity in multiple chains, Met1-, Lys63-, Lys48-, and Lys27-linked chains, as well as mono-ubiquitination, while cooperating with E2 to synthesize Lys-linked or Met1-linked chains. The Ub tag attached to a certain substrate, which is preferably achieved through the cooperation of specific E2/E3 enzyme pairs, represents a complex yet specific message encoded by the cell (Kim and Huibregtse, 2009; David et al., 2011; Turek et al., 2018). Interestingly, this is achieved by Ub receptors which are equipped with one or more UBDs (Mattern et al., 2019). Ubiquitin recognition achieved by UBDs can translate written code into specific outcomes. There are more than 20 families of UBDs that bind to different patches on Ub surrounding hydrophobic patches, either lle44 or lle36 (Dikic et al., 2009; Hendriks et al., 2018). Moreover, UBDs are able to sense unique 3D conformations of distinct chain types, which are associated with diverse biological activities, meaning that this small motif-containing protein can help recognize versatile signals and affect the desired effect (Lee et al., 2006; Penengo et al., 2006; Reyes-Turcu et al., 2006; Bomar et al., 2010). In addition, ubiquitin-interacting proteins which served as “decoders” of the ubiquitin message, participated in the downstream regulation of the ubiquitinated substrate (Heideker and Wertz, 2015; Leznicki and Kulathu, 2017; Mevissen and Komander, 2017). These “decoders” may specifically reverse the ubiquitination process or function as receptors for the transfer of the targeted substrate toward downstream signaling components and/or subcellular compartments (Hu et al., 2002; Lin et al., 2008; Komander et al., 2009). To date, 55 ubiquitin specific proteases (USPs), 14 ovarian tumor DUBs (OTUs), 10 JAMM family DUBs, 4 ubiquitin C-terminal hydrolases (UCHs) and 4 Josephin domain DUBs have been identified. Encoding and decoding ubiquitin codes are responsible for all levels of epigenetic changes, and by changing substrate protein activities, they can also activate and repress effects on gene transcription depending on their target proteins and the ubiquitin chain types, all of which are connectively related to the process of tumor proliferation (Kim and Baek, 2006; Zheng et al., 2008; Fradet-Turcotte et al., 2013; Yeh et al., 2018). Ubiquitin code signaling is frequently dysregulated in numerous cancer types and can function as a tumor suppressor or tumor promoter, suggesting a potential target for cancer therapy (Loch and Strickler, 2012; Choudhry et al., 2018; Emanuelli et al., 2019).

### 1.4 Physiological Functions of Non-proteolytic Poly-Ubiquitin Chains

The simplest version of ubiquitination or mono-ubiquitination confers non-degradative activities including protein localization (Yang et al., 2017), endocytosis (Shih et al., 2002; Windheim et al., 2008), trafficking, DNA repair (Xie et al., 2014; Whiteaker et al., 2018), autophagy (Chen S. et al., 2017; Zheng et al., 2020; Leng et al., 2021) and chromatin remodeling (Cole et al., 2021). When mono-ubiquitination is further modified, multiple lysine residues of the substrate are yielded to induce multi-mono-ubiquitination. Emerging investigations have found that this process connects ubiquitin with subcellular trafficking (Yin et al., 2010; Cooray et al., 2011) and immune-suppressive functions (Zhu et al., 2018). As for poly-ubiquitination, Lys48-linked poly-ubiquitination leads to the degradation of substrates (Komander and Rape, 2012). In contrast, Lys63-linked poly-ubiquitination exerts critical signaling functions in regulating protein stability, including nuclear factor κB (NF-κB) signaling (Syed et al., 2006; Wu Z. et al., 2014; Gallo et al., 2014), endocytosis (Galan and Haguenauer-Tsapis, 1997), DNA damage responses, and immune responses (Wu and Karin, 2015; Hrdinka et al., 2016; Liu et al., 2017; Paul and Wang, 2017). Emerging experiments have shown that incorrect regulation of cellular processes (either tumour inhibitors or promoters) contributes to cancer pathogenesis and progression (Shirane et al., 1999). Additionally, the cellular functions of atypical ubiquitin linkages (except Lys11-linked ubiquitin) are supposed to be non-degradable (Kulathu and Komander, 2012; Iwai, 2014, 2015; Meza Gutierrez et al., 2018) and in most cases, activities involved in Lys63-linked poly-ubiquitin chains are also considered to be non-degradable (Liu et al., 2015).

Of note, other chains also regulate specific physiological functions: Lys6-linked poly-ubiquitin was supposed to be indirectly linked to DNA damage response with the help of heterodimeric ubiquitin E3 ligase BRCA1–BARD1 (Wu-Baer et al., 2003; Wu-Baer et al., 2010). Linear (M1) chains can regulate NF-κB activation (Behrends and Harper, 2011; Chen et al., 2015; Borghi et al., 2018), whereas Lys11 linkage acts as a powerful degradation signal in heterotypic ubiquitin conjugates (Locke et al., 2014; Mevissen et al., 2016) and lys27 linkage can prompt mitochondrial depolarization and mediate translocation of the E3 ligase Parkin, which accumulates Lys27-linked linkages on mitochondrial protein voltage-dependent anion-selective channel protein1 (VDAC1). This exact Lys27-linked translocation leads to Parkinson’s disease in the presence of Parkin (Geisler et al., 2010; Glauser et al., 2011). Lys27 linkage has also been demonstrated to be related with the DNA damage response and innate immune response (Xue et al., 2018). The propagation of Wnt/β-catenin signaling through Lys29-or Lys11-linked ubiquitin chains is closely associated with cancer pathogenesis and is involved in protein ubiquitination at multiple levels (Hay-Koren et al., 2011). Several studies have reported the functions of both Lys29-and Lys33-linked chains in the regulation of AMPK-related protein kinases (Al-Hakim et al., 2008), and the Lys33 linkage is negatively regulated by T-cell antigen receptors (TCRs), which indirectly affect cellular activities in tumors (Huang et al., 2010). In this review, we will focus on recent progresses in non-degradable ubiquitin chains in tumorigenesis and the essential proteins involved in non-proteolytic ubiquitination.

## 2 The Roles of Non-Proteolytic Ubiquitination in Tumorigenesis

**FIGURE 2:**
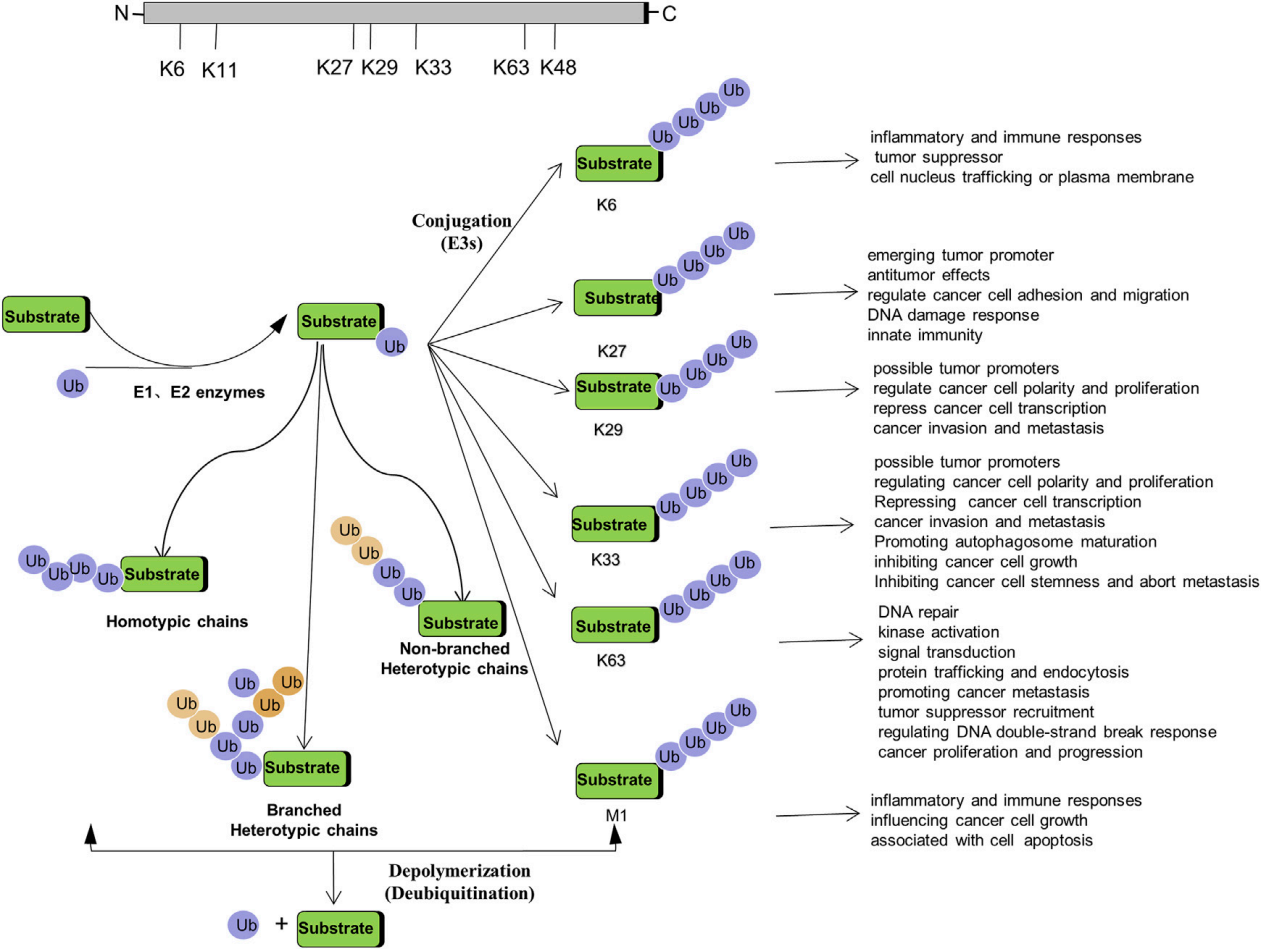
Regulation of Tumorigenesis by Non-proteolytic Ubiquitination: Linked through Lys6, Lys27, Lys 29, Lys 33, Lys 63, M1 poly-ubiquitination.

Non-proteolytic ubiquitination, including both mono-ubiquitination and poly-ubiquitination (mainly K6-, K27-, K29-, K33-, K63-, and M1-linked poly-ubiquitination), has become key regulators in a variety of cancers. How they function as signaling entities in the pathogenesis and progression of cancer will be beneficial to gain an in-depth understanding of ubiquitination (Figure 2).

### 2.1 Mono-Ubiquitination

Mono-ubiquitination has been suggested to be even more dynamic than previously thought, and its functions have been deciphered by various ubiquitin-binding proteins. It has been demonstrated that the cellular functionality of ubiquitin is mediated by mono-ubiquitin and/or poly-ubiquitin. In fact, mono-ubiquitination is associated with tumorigenesis, not limited to membrane transportation, endocytosis, receptor internalization, degradation in lysosomes, and protein reprocessing (Haglund et al., 2003). BMI1 interacts with histone H2A through mono-ubiquitination, repressing multiple genes, such as INK4A/ARF, which function in the pRb and p53 pathways, thereby facilitating cancer progression (Lin et al., 2015). Interestingly, in the presence of UbE2E1, PRC1 catalyzes the mono-ubiquitination of H2A, contributing to cancer cell proliferation (Wheaton et al., 2017). Mono-ubiquitination is also involved in the process of USP22 regulating histone H2B and exhibits both oncogenic and tumor-suppresser roles in cancer development (Jeusset and McManus, 2017). In addition, reversible mono-ubiquitination activity plays an essential role in balancing TGF-β/SMAD signaling, which is involved in cancer initiation and progression (Xie et al., 2014). Mono-ubiquitination also regulates forkhead box O (FOXO) proteins, which control specific gene expression programs that are vital for slowing the onset of cancer in aging individuals (Greer and Brunet, 2008). Strikingly, FANCL cooperates with, UBE2T and catalyzes mono-ubiquitination, which participated in the regulation of Fanconi Anemia pathway, leading to chromosome instability and promoting tumorigenesis (Machida et al., 2006; Hodson et al., 2014; Miles et al., 2015; Sun et al., 2020; Wang S. et al., 2021) (Table 1, 2).

**TABLE 1:** Summary of the combined E2/E3 enzymes in Tumorigenesis.

E2	Alias	Accompanied E3	Linkage	Phenotypic Characteristics	Neoplastic Implications	Substrate	Mechanism Summary
UBE2N	UBC13	TRAF2/TRAF6?	K63	preventing tumor formation and metastasis	modulating breast cancer metastasis	NEMO?	UEV1A, together with Ubc13, promote breast cancer metastasis through Lys63-linked polyubiquitination of target proteins and NF-кB-mediated MMP1 expression (Wu et al., 2014b)
UBE2N	UBC13	TRAF6	K63	DNA damage repair and protein kinase activation	metastatic spread and lung colonization by breast cancer cells	p38	Ubc13 catalyzes K63-linked proteins, accompnied by TAK1-p38 activation, whose activity is essential for breast cancer metastasis (Wu et al., 2014a)
Ubc13:Uev1A/Uev2	UBE2N:UBE2V1/Uev2	RNF8	K63	DNA damage repair and cytokinesis	genomic instability in adult T-cell leukemia (ATL)	TAK1?IKK?	Ubc13/Uev1A and RNF8 interact with each other and generate K63-pUb, which is recognized by Tax, stimulating TAK1 and IKK activation (Ho et al., 2015)
Ubc13	UBE2N	TRAF6	K63	activating NF-κB signaling	elicit anti-tumour responses	RANK?	STAT3 negatively regulates Ubc1 involving K63-linked ubiquitination, and suppress pro-inflammatory cytokines by modulating NF-κB signaling (Zhang et al., 2014)
Ubc13	UBE2N	RNF8	K63	DNA double-strand break (DSB) responses	BRCA1 Tumor Suppressor Recruitment	histone	RNF8 stimulates Ubc13-dependent Lys-63 ubiquitination, which is pivotal for DNA damage response and recruitment of BRCA1 (Hodge et al., 2016)
Ubc13	UBE2N	TRAF6	K63	innate and adaptive immunity	osteoclast differentiation	TRAF6 (autoubiquitination)	Ubc13/Uev1A interacts and binds to the active RING domain of TRAF6, which is essential for the formation of Lys63-linked pUb, thus triggering NF-κB activation and osteoclast differentiation (Lamothe et al., 2008)
Ubc13/Uev1A	UBE2N/UBE2V1	TRAF6	K63	activating NF-κB signaling	osteoclast differentiation	TRAF6 (autoubiquitination)	TRAF6 in combination with Ubc/Uev1A catalyzes TRAF6 auto-ubiquitination through Lys63-linked poly-Ub chains, which controls NF-κB signaling and osteoclast differentiation (Lamothe et al., 2007b)
Ubc13/Uev1A	UBE2N/UBE2V1	TRAF6	K63	spontaneous osteoclast differentiation		TRAF6 (auto-ubiquitination)	TRAF6 interacts with Ubc13/Uev1A, facilating Lys-linked quto-ubiquitination of TRAF in a RING domain-dependent fashion, and modulating downstream NF-κB signaling (Lamothe et al., 2007a)
UBE2O	UBE2O	TRAF6	K63	activating NF-κB signaling	modulating NF-κB signaling associated cancers	TRAF6 (auto-polyubiquitination)	UBE2O negatively regulates the recruitment of TRAF6, inducing TRAF6 auto-ubiquitination through binding to K63 residue, and subsequently prevents NF-κB activation by the IL-1R/TLR complex (Zhang et al., 2013b)
UBE2T	UBE2T	FANCL	monoubiquitination	maintenance of chromosome stability	disrupting DNA repair pathways	FANCD2	In the presence of FANCL, UBE2T stimulates monoubiquitination of FANCD2, which is vital for disrupting abnormal chromosomes and efficient DNA damage repair (Machida et al., 2006)
UBE2T	UBE2T	FANCL	automonoubiquitination	maintenance of chromosome stability	disrupting DNA repair pathways	UBE2T	Automonoubiquitination of UBE2T inhibits own conjugation activity (Machida et al., 2006)
UBE2T	UBE2T	FANCL	monoubiquitination	DNA repair	leading to leukemia and bone marrow failure	FANCD2	FANCL interacts with UBE2T in an ELF-domain-dependent fashion, which regulates DNA damage-induced FANCD2 monoubiquitination (Miles et al., 2015)
UBE2T	UBE2T	FANCL	monoubiquitination	DNA interstrand crosslink repair	genomic instabilities	FANCD2	FANCL specifically interacts with UBE2T, leading to FANCD2 ubiquitination, which is involved in Fanconi Anemia pathway (Hodson et al., 2014)
RAD6	UBE2B	in absence of E3	K63?	promoting DNA repair	promoting recurrence and metastasis in ovarian cancer	β-catenin	Rad6 facilitates DNA repair and stem cell gene transcription, through mediating H2B ubiquitination (Somasagara et al., 2017)
RAD6B	UBE2B	in absence of E3	K63	DNA repair and mutagenesis	h-catenin modification in breast cancer	β-catenin	Rad6B ubiquitinates b-catenin through K63-linked ubiquitination, which regulates transcriptional activity in breast cancer (Shekhar et al., 2008)
UBE2B	UBE2B	BRE1	monoubiquitination	promoting the G1-S transition and cell proliferation	promoting G1-S transition and cell proliferation	H2B	UBE2B modulates CCND1 transcription level by regulating the levels of H2B monoubiquitination, promoting cell cycle progression and proliferation (Cai et al., 2014)
Ube2w	UBE2W	RNF4	monoubiquitination	DNA damage repair	potential prostate, breast and lung cancer target	SUMO	Ube2w associated with RNF4, mediating mono-ubiquitination of SUMO chains. Those chains can be further ubiquitinated through K63 chains in response to DNA damager (Maure et al., 2016)
Ubc13	UBE2N	TRAF6?	K63	Autoimmunity and aberrant T cell activation	modulating NF-κB associated cancer	IKK	Ubc13 conjugates K63-linked ubiquitin chains involving Ubc13-IKK signaling axis, which have a robust evidence in regulating T cell function (Chang et al., 2012)
UBC13	UBE2N	Bcl10	K63	activating NF-kappaB pathway	modulating NF-κB associated cancer	NEMO	UBC13 is dependent in Bcl10 modulating NEMO lysine-63-linked ubiquitination, and subsequent NF-kappaB activation (Zhou et al., 2004)
UBE2N	UBE2N	MEKK1	K63	embryonic survival	promoting ES-cell differentiation and tumour formation	TAB1	Together with UBE2N, MEKK1 could tag TAB1 with Lys63-linked poly-Ub, promoting ES-cell differentiation and tumourigenesis (Charlaftis et al., 2014)
UbcH6	UBE2E1	TRAF4	K63	DNA damage	overcome chemoresistance in colorectal cancer	CHK1	UbcH6 combined with TRAF4, which is critical for CHK1 K63-linked ubiquitination and essential for cell proliferation, colony formation (Yu et al., 2020b)
UBE2T	UBE2T	RNF8	monoubiquitination	facilitating cell cycle arrest activation	conferring HCC radioresistance	H2AX	UBE2T/RNF8 complex, monoubiquitinated H2AX/γH2AX, facilitating cell cycle arrest activation, thus inducing HCC radioresistance (Sun et al., 2020)
Ubc13:Uev1A/Uev2	UBE2N:UBE2V1/UEV2	RNF8	K63	DNA damage repair and cytokinesis	genomic instability of ATL cells	NEMO and TAB2/3	RNF8and Ubc13:Uev1A/Uev2assemble K63-pUb chains on NEMO and TAB2/3 respectively, allowing TAK1 and IKK activation (Ho et al., 2015)
UbE2E1		PRC1	monoubiquitination	maintenance of stem cell proliferation	promoting cancer cell proliferation	H2A	UbE2E1 interacts with PRC1 complex, catalyzing monoubiquitination of H2A (Wheaton et al., 2017)
Ubc13	UBE2N	TRAF6	K63	regulating immune signaling	NF-κB signaling related cancer		Ubc13, together with TRAF6, mediates K63-linked polyubiquitin signaling pathway, including NF-κB signaling (Lenoir et al., 2021)
Uev1A/Ubc13	UBE2N/UBE2V1	TRAF6	K63	regulating AKT signaling pathway	promoting breast cancer cell migration and EMT signaling	AKT	Uev1A/Ubc13 interact with TRAF6, ubiquitinates AKT with K63-linked ubiquitination, which is required for AKT activation, promoting cell migration and EMT in breast cancer (Niu et al., 2021)
UBE2T		FANCL	monoubiquitination	involving in FA pathway-induced chromosome instability	functions in cancer predisposition	ID	UBE2T/FANCI-FANCD2 complex remodeling the ID-DNA complex, preventing clamp opening after monoubiquitination (Wang et al., 2021a)
UbcH6		NEDD4	K63	regulating cell-cell adhesion, mechanosensing and autophagy	involving in angiogenesis and tumor growth	IGPR-1	NEDD4 and UbcH6 are involved in the K63-linked ubiquitination of IGPR-1, regulating different cellular activities, such as cell adhesion, autophagy, mechanosensing, angiogenesis and tumor growth (Sun et al., 2021)
Ubc13	UBE2N	RNF213	K63	angiogenic activity	regulating cell mobility and invasion		RNF213 interacts with Ubc13 and promotes its own autoubiquitination, controling inflammatory responses and angiogenic activities (Habu and Harada, 2021)

**TABLE 2:** Summary of the Identified DUBs Involving in Tumorigenesis.

Type	DUBs	Linkage	Phenotypic Characteristics	Neoplastic Implications	Substrate	Mechanism Summary
OTU	TRABID (ZRANB1)	K63	stem cell self-renewal or differentiation	Wnt-induced transcription in colorectal cancer cell	APC	Trabid preferentially binding to K63-linked ubiquitination chains, which is required for Wnt-induced transcription (Tran et al., 2008)
OTU	TRABID (ZRANB1)	K29, K33	inhibiting autophagy flux	Promoting autophagosome maturation and inhibiting hepatocellular carcinoma growth	UVRAG	ZRANB1 precisely cleaves K29 and K33-linked polyubiquitin chains from UVRAG, regulating autophagy system (Feng et al., 2019)
OTU	OTUD1	K33	restricting the TGF-β signaling	inhibiting breast cancer stemness and metastasis	SMAD7	OTUD1 directly deubiquitinates the SMAD7, shuts off TGF-β signals, thereby suppressing metastasis in breast cancer (Zhang et al., 2017)
OTU	OTUD1	K63	regulating organ growth, tissue regeneration	Regulating tumorigenesis, cancer cell survival and chemoresistance	YAP	OTUD1 cleaves K63-linked poly Ub from YAP, which is assembled by SKP2 E3 ligase, regulating tumorigenesis (Yao et al., 2018)
OTU	OTUD1	K63?	suppressing colony formation and increasing apoptosis	arresting cell growth and inducing apoptosis	p53	OTUD1 interacts with and stabilizes p53. Its overexpression significantly suppress colony formation, and increases apoptosis (Piao et al., 2017)
OTU	OTULIN	M1	activating NF-κB and promoting pro-inflammatory cytokines and restricting bacterial proliferation	NF-κB signaling associated cancers	cytosolic *salmonella*	OTULIN dissolutes linear Ub chains on cytosolic S, resulting in ultimately NF-κB activation (van Wijk et al., 2017)
OTU	OTULIN	M1	regulating NF-κB signaling and sensitizing cell death	NF-κB signaling associated cancers	RIPK1	OTULIN interacts with LUBAC, balancing Met1-polyUb chains, thereby regulating NF-κB signaling (Keusekotten et al., 2013)
OTU	OTUD1	K63?	decreases cell proliferation and increases cell apoptosis	regulating tumor-suppressor p53	p53	OTUD1 interacts with and deubiquitinates p53, regulates p53 stability and activity (Piao et al., 2017)
OTU	A20	K63	NF-κB transcriptional activity-mediated cell death and chronic inflammation	NF-kB signaling-related cancers	RIP	A20 erases K63-linked ubiquitin chains from RIP, and it also polyubiquitnate RIP with K48-linked ubiquitin chains in a carboxy-terminal-domain-dependent manner, which downregulating NF-kB signalling, (Wertz et al., 2004)
OTU	A20	K63	downregulating NF-kB pathway	NF-kB signaling-related cancers	TRAF6/RIP	A20 display dual ubiquitin-editing functions, mediating both non-proteolytic Lys63-linked ubiquitin chains and degradative Lys48-linked ubiquitin chains, thus regulating NF-κB activities (Lin et al., 2008)
OTU	OTUD7B	K63	regulating mTORC2 signalling, thus relating to cell growth and metabolic disorders	activates Akt signaling and Kras-driven lung tumorigenesis *in vivo*	GβL	OTUD7B and TRAF2 regulate stability of GβL, which plays critical roles in mTORC2 signaling (Wang et al., 2017a)
UCH	BAP1	monoubiquitination	inhibiting cell apoptosis	mediating tumor suppression in both mice and humans	H2A	Inactivation of BAP1 causes apoptosis through regulating H2A monoubiquitination, regulating tumor suppression (He et al., 2019)
USP	USP4	K63	activating inflammation and immune response	inhibiting TNFα-induced cancer cell migration	TRAF2/TRAF6	USP4 negatively regulates the TRAF2- and TRAF6-stimulated NF-κB activation, and inhibits cancer cell migration (Xiao et al., 2012)
USP	CYLD	K63	regulating NF-κB-mediated inflammation	associating the development of head and neck squamous cell carcinomas	NEMO	TRAF3/CYLD complex regulate NF-κB transcriptional level, which is associated with head and neck squamous cell carcinomas with HPV infection (Chen et al., 2017b)
USP	CYLD	K63/M1	regulating Innate Immune Signaling	tumor suppressor	RIPK2	CYLD counteracts Met1-Ub and Lys63-Ub conjugated to Ripk2, and this deubiquitinase activity plays an important role in innate immune regulation (Hrdinka et al., 2016)
USP	USP8	K63	DNA damage response	genomic instability in cancer	BRIT1	USP8 rescues BRIT1 from K63 ubiquitin and regulates its recruitment to DNA double-strand break sites (Ge et al., 2015)
USP	USP10	K63	controlling cell cycle	promoting proliferation of t chronic myeloid leukemia cells	Bcr-Abl	SKP2 acts as co-regulator of K63-linked ubiquitination of Bcr-Abl for its activation. While USP10 deubiquitinates and stabilizes SKP protein levels and amplifies Bcr-Abl activation in chronic myeloid leukemia cells (Liao et al., 2019)
USP	USP20	K63	negatively regulating inflammation, cell proliferation, and apoptosis	promoting adult T cell leukemia (ATL) development	Tax	USP20 targets and deubiquitinates TRAF6 and TAX, negatively regulating NF-κB signaling (Yasunaga et al., 2011)
USP	USP17	K63	enhancing inflammation and promoting macrophage recruitment	promoting lung cancer growth	cIAP1/2	USP17 interacted with and disrupted the TRAF2/TRAF3 complex through reducing K63-linked ubiquitination of TRAF2 and TRAF6. This activity positively drives stemness and inflammation in lung cancer (Lu et al., 2018)
USP	CYLD	K63	regulating inflammation	promoting tumor growth	TAK1	Itch-Cyld complex sequentially cleaving K63-linked ubiquitin chain on Tak1 thus terminating the inflammatory response (Ahmed et al., 2011)
USP	CYLD	K63	negative regulate the NF-κB pathway	tumor suppressor	E6	HPV E6 suppresses the CYLD under hypoxic conditions, promoting unrestricted NF-κB activation and allowing for malignant progression of tumors (An et al., 2008)
USP	CYLD	K63	controling inflammation	inhibiting tumor formation	Bcl-3	Cyld erases K63-linked polyubiquitin chains from Bcl-3, inactivating NF-κB signaling (Massoumi et al., 2006)
USP	CYLD	K63	controls survival and inflammation	inhibiting tumor cell Proliferation	TRAF2	Cyld regulates inflammation through deubiquitinating TRAF2 and blocking NF-κB pathway (Massoumi et al., 2006)
USP	USP14	K63	inflammation	acute colitis and colitis-associated colon cancer development	p100/p52	TRAM14 recruits USP14 to cleave K63-linked ubiquitin chains of p100/p52, regulating NF-κB-mediated autophagy and innate immunity. (Chen et al., 2020)
USP	USP1	K63	regulating macroautophagy/autophagy	affecting breast cancer cell growth	ULK1	USP1 modulates ULK1 K63-linked deubiquitination, and regulates autophagy, also affects breast cancer cell growth relying on autophagy (Raimondi et al., 2019)
USP	USP1	K63	Double-strand breaks (DSBs)	potential tumour suppressor	histones	USP1 actively destorys K63-linked poly-ubiquitin chains on histones. And its recruitment to damage sites has a close link with genome stability and double-strand breaks (Ha et al., 2017)
USP	USP34	K63	genome stability maintenance	promoting ES-cell differentiation and tumour formation	H2A	USP34 stabilizes RNF168, recruiting repair proteins at DSBs, which, is critical for genome stability (Sy et al., 2013)
USP	CYLD	K63/M1	DNA damage-induced apoptosis	enhancing sensitivity to chemodrug in cancer cells	NEMO	CYLD downregulates K63-linked and linear ubiquitination of NEMO, promoting apoptosis (Niu et al., 2011)
USP	CYLD	K63	activating the cell death pathway	regulating ATLL cell death	RIPK1	CYLD erases k63-linked ubiquitin chains from RIPK1, which activates the cell death pathway and activates CYLD and RIPK1-dependent tumor cell death in ATLL (Xu et al., 2020)
USP	USP38	K63	restoring genome stability	regulating cancer cell response to genotoxic insults	HDAC1	USP38 preferentially removed the K63-linked ubiquitin chains from HDAC1, regulating genomic stability (Yang et al., 2020)
	UBQLN4	K63/K48	homologous recombination repair	predictor of poor survival in various cancer entities	MRE11	Overexpression of UBQLN4 represses homologous recombination activity through inhibiting MRE11 ubiquitination, thus presenting close relationship with survival rates in various cancer (Jachimowicz et al., 2019)
OTU	ZRANB1	K29/K33	promoting autophagosome maturation	inhibiting cell growth in hepatocellular carcinoma	UVRAG	ZRANB1 specifically cleaves SMURF1-induced K29 and K33-linked polyubiquitin chains from UVRAG, regulating autophagosome maturation and HCC growth (Feng et al., 2019)
JAMM	POH1	K63	double-strand DNA break responses, embryonic stem cell differentiation	promoting tumour formation in human hepatocellular carcinomas (HCCs)	E2F1	POH1 binds to and stabilizes the E2F1, upregulating Survivin and FOXM1 protein levels, accompanied by accelerating tumor growth (Wang et al., 2015)
USP	USP30	K6	regulating mitophagy and neurodegenerative disease	functions in hepatocellular carcinoma		USP30 specifically cleaves the Lys6 linked ubiquitin chains, regulating mitophagy, apoptosis and tumorigenesis
OTUD1	OTUD1	K63	suppressing intestinal inflammation	NF-kB signaling-related cancers	RIPK1	OTUD1 prefercially cleaves K63-linked polyubiquitin chains from RIPK1, inhibiting colonic inflammation and NF-κB signaling
USP	CYLD	K63	regulating ERK activation	regulating cancer cell growth, proliferation, migration	ERK1/2	CYLD cleaves K63-linked ubiquitination mediated by TRIM1, regulating ERK signaling and the assocaited cancer development
USP	USP10	K63		inhibiting NSCLC cell proliferation and migration	PTEN	USP10 suppress NSCLC cell proliferation and migration through abolishing PTEN from K63-linked polyubiquitination mediated by TRIM25
OTU	A20	K63	anti-inflammatory effects	tumor suppressor	TBK1	A20 inhibits TBK1 activation through reducing K63-linked ubiquitination of Nrdp1, regulating inflammation

### 2.2 Linear (M1) Linkage

#### 2.2.1 The LUBAC Complex Encodes the M1 Linkage

Emerging evidences connect Met1-linked ubiquitin chains to NF-κB signaling, which enables physiological regulation of inflammation and immune responses (Emmerich et al., 2011; Borghi et al., 2018). Indeed, mutations and deficiencies involved in the formation and dissolution of Met1-linked poly-ubiquitin chains have been extensively illustrated in immune-related disorders (Tokunaga and Iwai, 2012; Fiil et al., 2013; Fiil and Gyrd-Hansen, 2014). Until now, linear ubiquitin chain assembly complex (LUBAC) is the only known E3 ubiquitin ligase assembling this type of chain (Kirisako et al., 2006; Walczak et al., 2012). The multi-subunit E3 ligase comprises catalytically active hybrid organic-inorganic perovskite (HOIP, also known as RNF31) and two adaptor proteins, HOIP1L (also known as RBCK1) and SHANK-associated RH domain-interacting protein (SHARPIN) (Tokunaga et al., 2011). Both HoIL-1 and SHARPIN can co-operate with HOIP, which might be a central part of the LUBAC complex (Ikeda et al., 2011; Elton et al., 2015). The HOIP orthologue, linear ubiquitin E3 ligase (LUBEL), modifies Kenny with M1-linked linear ubiquitin chains in *Drosophila* and is indispensable for inflammatory responses by activating Imd pathway (Aalto et al., 2019). Additionally, HOIP, with the help of cIAP1, is recruited to the linear ubiquitination of the TNFR2 signaling complex and activates canonical NF-κB, thereby facilitating cancer progression (Borghi et al., 2018). Multiple investigations found that HOIP, presumably through M1-linked ubiquitination, was proved to be connected with sorts of malignancies, including breast and prostate cancer (Guo et al., 2015; Zhu et al., 2016). Furthermore, previous experiments highlight that HOIL-1 interacts with HOIP, which adds a Mi-linked poly-ubiquitin chain to specific NF-κB signaling proteins, suggesting its link to a diversity of immune disorders, antiviral signaling (Elton et al., 2015), iron and xenobiotic metabolism (Elton et al., 2015), apoptosis (Emmerich et al., 2011; Lewis et al., 2015; Sasaki and Iwai, 2015), and cancer (Queisser et al., 2014; Taminiau et al., 2016). Another member of the LUBAC family, SHARPIN, is a novel component of the LUBAC complex. Spontaneous mutation of this tiny gene led to dysregulation of the NF-κB signaling pathway through linear ubiquitinate of NEMO (the key modulator of NF-κB). This systematic linear ubiquitination is obvious and can induce immune system disorders in SHARPIN-deficient mice (Tokunaga et al., 2011). Interestingly, SHARPIN-containing complexes can also interact with NEMO to activate NF-κB pathway (Ikeda et al., 2011). Moreover, NEMO was identified to be modified by LUBAC, generating M1-linked chains that were recognized by the UBAN domain of NEMO, causing conformational changes in the intertwined helices of NEMO dimers (Haas et al., 2009; Belgnaoui et al., 2012; Tokunaga and Iwai, 2012; Noad et al., 2017). NF-κB signaling regulates human cellular activities in different ways and is balanced by ubiquitination and deubiquitination (Adhikari et al., 2007; Lork et al., 2017) (Table 2).

#### 2.2.2 OTULIN Dissembles M1-Linked Poly-Ubiquitin Chains

OTULIN, a methionine 1 (M1)-specific deubiquitinase (DUB), is a rare member of the OTU family of DUBs. Its proximal ubiquitin moiety cannot break down isopeptide linkages of ubiquitin chains, but can efficiently cleave peptide bonds present in the linear chains (Keusekotten et al., 2013; Rivkin et al., 2013). OTULIN presents negative regulation in the cellular process of immune homeostasis and inflammation (Damgaard et al., 2019). Depletion of OTULIN resulted in an increase in the formation of linear Ub chains and demonstrated proteasome dysregulation as the cause of NF-κB positive activation, which in turn restricts bacterial proliferation (Takiuchi et al., 2014; van Wijk et al., 2017). Moreover, the deficiency of OTULIN led to the inability to remove M1-linked poly-ubiquitin signals, which are typically conjugated by the LUBAC, resulting in LUBAC degradation and dysregulation of TNF signaling and cell death (Tokunaga, 2013; Damgaard et al., 2016). Notably, the function of LUBAC is controlled by cylindromatosis (CYLD), which interacts with the LUBAC core subunit HOIP to generate Met1-linked ubiquitin. However, this interaction can be weakened by the Met1-Ub-specific deubiquitinase OTULIN. In addition, through the deubiquitinase function of OTULIN, LUBAC can regulate Met1-Ub to ensure an advisable response to innate immune activity (Elliott et al., 2016; Hrdinka et al., 2016). The CYLD/TRAF3 complex has also been reported to regulate NF-κB-mediated inflammation and interferon signaling, which defines a subset of head and neck cancers that harbor human papillomavirus (Chen T. et al., 2017). Notably, accumulating evidence has manifested that linear ubiquitin chains play essential roles in ensuring appropriate activity of inflammatory responses and innate immune signaling (Tokunaga and Iwai, 2012; Tokunaga, 2013; Jing et al., 2017). The linear ubiquitination of cFLIP is directly induced by RNF31, a catalytic subunit of LUBAC, at Lys-351 and Lys-353, contributing to TNFα-induced apoptosis, thereby protecting cells from apoptosis (Tang et al., 2018) (Table 3).

**TABLE 3:** Summary of the E3 enzymes in Tumoregenesis.

E3	Linkage	Phenotypic Characteristics	Neoplastic Implications	Substrate	Mechanism Summary
RNF8	K63	regulating DNA double-strand break responses	BRCA1 Tumor Suppressor Recruitment	histone	RNF8 interact with Ubc13, generating K63-linked ubiquitin chains on histone, which positively regulate DNA double-strand break and BRCA1 recruitment (Hodge et al., 2016)
RNF8	K63	DNA damage repair and cytokinesis	genomic instability of ATL cells	Tax	Stimulated by Tax, RNF8 and Ubc13:Uev1A function together, generating K63-pUb chains, whichactivated TAK and NF-κB signaling (Zhi et al., 2020)
HOIL-1	M1	immune regulation	NF-kB activation in cancers	NEMO	HOIL-1 modifies NF-κB core proteins with linear ubiquitin chains, regulating NF-κB pathway signaling (Elton et al., 2015)
CHIP	K6	suppressing of cell death		FADD	CHIP triggers K6-linked polyubiquitylation of FADD, leading to the suppression of cell death (Seo et al., 2018)
BRCA1	K6	DNA damage response	tumor suppressor	BRCA1 autoubiquitination	UBXN1binds to BRCA1 active site and decorate it with K6-linked polyubiquitin chains in a UBA-domain-dependent manner and BRCA1/BARD1 complex is regulated by the ubiquitinate status of BRCA1 (Wu-Baer et al., 2010)
BRCA1	K6	DNA repair, transcrip- tional regulation, and cell cycle checkpoint control	tumor suppressor	BRCA1 autoubiquitination	BRCA1 mediates autoubiquitination by conjugating to K6-linked polymers, which impart cellular properties (Wu-Baer et al., 2003)
BRCA1	K6	DNA double-stranded breaks repair	tumor suppressor	might be BRCA1 autoubiquitination?	BRCA1 recruits its autoubiquitination at DNA damage sites, which is dependented on K6-linked linkage. BRCA1:BARD1 enzyme activity is regulated by BRCA1 ubiquitin status. (Morris and Solomon, 2004)
BRCA1	K6	regulating DNA repair, transcriptional leves, cell cycle and cell apoptosis	tumor suppressor	BRCA1 autoubiquitination	BRCA1-BARD1 regulate BRCA1 autoubiquitination by preferentially mediating K6-linked polyubiquitin chains (Nishikawa et al., 2004)
Hectd3	K27, K29	leading to NF-kB activation	NF-κB associated cancer	Malt1	Hectd3 promotes K27 and K29 polyubiquitination on Malt1, regulating autoimmunity and other Th17-related diseases (Cho et al., 2019)
WWP1	K27	suppressing the dimerization, membrane recruitment	restoring tumor-suppressive activity	PTEN	WWP1 triggers K27-linked polyubiquitination of PTEN to regulate subcellular localization cancer susceptibility syndromes (Lee et al., 2019c)
TRAF4	K27, K29	facilitating immune cell migration	promoting cancer cell invasion	TrkA	TRAF4 promotes K27 and K29-linked ubiquitin linkages on TrkA, facilitating prostate cell invasion (Singh et al., 2018)
RNF4	K63	DNA damage repair	potential prostate, breast and lung cancer target	Trim5α	Ube2w interacts with RNF4, promoting monoubiquitination of SUMO chains, which are further modified to form K63-linked ubiquitin chains (Maure et al., 2016)
RNF8	K63	DNA damage response	breast cancer predisposition	H2A/H2AX	RNF8 activated with Ubc13, promoting K63-linked polyubiquitin conjugation to histones H2A/H2AX, then contributing to breast cancer predisposition (Vuorela et al., 2011)
Skp2	K63	promoting survival and Akt-mediated glycolysis	restricting cancer cell progression	Akt	Skp2/SCF complex catalyzes K63-linked ubiquitination chains on Akt, which is required for glycolysis and cancer development (Chan et al., 2013)
Skp2	K63	controling cell cycle	promoting proliferation of chronic myeloid leukemia cells	Bcr-Abl	SKP2 triggers K63-linked ubiquitination of Bcr-Abl, regulating downstream signaling, and is vital for chronic myeloid leukemia development and progression (Liao et al., 2019)
RNF113A	K63	DNA repair	potentially associating with tumor progression	BRR2	RNF113A interacts with BRR2 through K63-linked polyubiquitin, mediating repairment of DNA alkylation damage (Brickner et al., 2017)
TRAF2	K63	mediating several cell growth and metabolic pathways	facilitating tumorigenesis	GβL	TRAF2 promotes K63-linked polyubiquitination of GβL, and regulats mTORC2 signalling, thus mediating several cell growth and metabolic pathways (Wang et al., 2017a)
TRIM37	mono-ubiquitination	regulating transcriptional repression	promoting transformation in breast cancer	H2A	TRIM37 mono-ubiquitinates histone H2A, thus associating with transcriptional repression (Bhatnagar et al., 2014)
RNF8/RNF168	K63	DNA double-strand breaks (DSBs)	mediating ATM-dependent carcinogenesis	H2A/H2AX	RNF8 and RNF168 combined together to catalyze K63-linked poly-Ub chains on H2A/H2AX, which is important for transcription and DNA double-strand breaks (Shanbhag et al., 2010)
HectH9	K63	regulating transcriptional activation and repression	tumor cell Proliferation	Myc	HectH9 recruits 63-linked polyubiquitin chains to Myc, modulating cell proliferation in various tumor cells (Adhikary et al., 2005)
Bcl10	K63	activating the NF-κB pathway	NF-κB associated cancer	NEMO	UBC13 and Bcl10function together inducing NEMO ubiquitination through lysine-63-linked ubiquitination, and subsequent NF-κB activation (Zhou et al., 2004)
RNF8	K63	DNA repair	tumour-promoting	probably hisotne H1	In p97–ATX3 activated conditions, RNF8 mediates K63-Ub at sites of DNA lesions, regulating genome instability, cell invasion and metastasis (Singh et al., 2019)
PARK2/Parkin	K33	fine-tune necroptosis and inflammation	tumor suppressor/inflammation-associated tumorigenesis	RIPK3	AMPK activated Parkin/RIPK3 complex through K33-linked polyubiquitination, which negatively regulates necroptosis and inflammation-associated tumorigenesis (Lee et al., 2019b)
TRAF6	K63/K27	maintaining nuclear genome integrity	promoting cancer progression	hDNA2	hTRAF6 catalyzes the K27- and K63-linked polyubiquitination of hDNA2, maintaining nuclear genome integrity and the associated cancer biology (Meng et al., 2019)
HectH9	K63	integrating glycolysis activation and apoptosis resilience	regulating tumor metabolism and cancer stem cell expansion	HK2	HectH9 catalyzes HK2’s K63-linked ubiquitination, regulating stem cell expansion and CSC-induced chemoresistance in prostate cancer (Lee et al., 2019a)
TRAF6	K63	regulating inflammation and immunity	promoting liver tumorigenesis and correlates with poor prognosis	HDAC3	TRAF6 ubiquitinates HDAC3 with K63-linked ubiquitin chains, regulating inflammation and malignant transformation and progression in HCC(Wu et al., 2020)
ITCH	K27	immune response	promoting proliferation and invasion of melanoma cells	BRAF	Activated ITCH maintains BRAF activity and subsequent MEK/ERK signaling through Lysine 27-linked ubiquitination, enhancing proliferation and invasion of melanoma cells (Yin et al., 2019)
TRAF6	K63	immunity	anti-tumor immunity in the cancer setting	FOXP3	TRAF6 bind to and facilitates Regulatory T cells (Tregs) activties through K63-linked ubiquitination at lysine 262, acting as aTreg-stabilizing regulator and playing crucial roles in immune control and anti-tumor immunity (Ni et al., 2019)
SMURF1	K29/K33	promoting autophagosome maturation	inhibiting cell growth in hepatocellular carcinoma	UVRAG	SMURF1 mediates K29 and K33-linked polyubiquitin chains on UVRAG, promoting autophagosome maturation and inhibiting hepatocellular carcinoma growth (Feng et al., 2019)
TRAF6	K63	NF-κB activation and autophagy activation	cancer cell migration, cell invasion	BECN1/TRAF6	PRDX1 negatively regulates TRAF6 ubiquitin-ligase activity, leading to NF-κB inactivation and autophagy activation (Min et al., 2018)
RNF8	K63	DNA double-strand break repair	regulating L3MBTL2 mutation in leukemia	L3MBTL2	MDC1 recruites L3MBTL2 to sites of DNA lesion and is then ubiquitylated by RNF8, promoting DNA DSB repair and regulating L3MBTL2-induced cancers (Nowsheen et al., 2018)
Itch	K63	regulating tissue patterning, stem cell maintenance	modulating medulloblastoma tumorigenesis	SuFu	Itch/β-arrestin2 complex mediates Lys63-linked polyubiquitylation on SuFu, thus controling Hedgehog signalling and medulloblastoma tumorigenesis (Infante et al., 2018)
FBXO32	K63	brain development	promoting tumorigenicity and metastasis in humans	CtBP1	FBXO32 directly ubiquitinates CtBP1 with K63-linked ubiquitin chains, and this interaction activity regulates downstream EMT signaling and is essential for tumor metastasis and brain development (Sahu et al., 2017)
Cullins	K29	promoting cell motility	modulating cell migration	hnRNP A1	SPSB1 catalyzes K29-linked polyUb chains on hnRNP A1, modulating cell migration and cell motility in EGF signaling (Wang et al., 2017b)
HUWE1	K63	preventing DNA damage accumulation	colonic tumour suppressor	Myc	Huwe1 mediates MYC transactivation activity via K63-linked ubiquitination, inhibiting accumulation of DNA damage and preventing tumour initiation especially in colonic cancers (Myant et al., 2017)
HectH9	K63	relating to embryonic lethal	promoting hypoxia-induced tumour progression	HAUSP(USP7)	HectH9 mediates K63-polyubiquitin chains conjugated to HAUSP. HAUSP then deubiquitinates HIF-1α, promoting hypoxia-induced tumour progression (Wu et al., 2016)
RNF8	K63	conferring chemoresistance	tumor-promoting function	Twist	RNF8 activates and ubiquitinate Twist, leading to subsequent EMT and CSC functions, thus exerting tumor-promoting functions such as cell migration and invasion (Lee et al., 2016)
FBXW7	K63	genome integrity	tumor suppressor	XRCC4	FBXW7 firstly phosphorylated by ATM and then it ubiquitylates XRCC4 via K63-linkage, promoting NHEJ repair, which is closely related to DSB and genomic stability (Zhang et al., 2016)
Trim7	K63	regulating proliferation and apoptosis	promoting Ras-mediated lung adenocarcinoma	RACO-1	Trim7 catalyzes Lys63-linked ubiquitination of RACO-1 in response to RAS signaling, and Trim7 overexpression increases lung tumour burden while knockdown of Trim7 reduces tumour growth in xenografts models (Chakraborty et al., 2015)
Skp2	K63	regulating energy metabolism, proliferation, apoptosis, and cell polarity	tumor growth in HCC	LKB1	Skp2-dependent activation of LKB1 through K63-linked Ubiquitination is essential for HCC tumor growth and related to poor survival outcomes (Lee et al., 2015)
TRAF6	K63	enhancing chemotherapeutic efficacy	promoting T-ALL progression	MCL1	IRAK1/4 signaling activated TRAF6, mediating K63-linked ubiquitination of MCL1, promoting T-ALL progression (Li et al., 2015)
PELI1	K63	maintenance of autoimmunity	promoting lymphomagenesis	BCL6	PELI1 specifically binds to BCL6 and induces lysine 63-linked ubiquitin chains on BCL6, promoting lymphomagenesis, modulating the maintenance of autoimmunity through TLR and TCR signaling (Park et al., 2014)
MEKK1	K63	embryonic survival	promoting ES-cell differentiation and tumour formation	TAB1	MEKK1 ubiquitylates TAB1 with Lys63-linked polyubiquitin in a PHD motif-dependent manner, inducing TAK1 and MAPK activation, which are crucial for ES-cell differentiation and tumourigenesis (Charlaftis et al., 2014)
TRAF4	K63	regulating immunity	driving Breast Cancer Metastasis	TβRI	TβRI-receptor TRAF4 interacts with each other, triggering Lys 63-linked TRAF4 polyubiquitylation and TAK1 activation, promoting cell migration and metastasis in breast cancer (Zhang et al., 2013a)
RNF8/RNF168	K63	maintaining genome stability	suppressing tumourigenesis	BLM	RNF8/RNF168, triggers BLM activation, leading to BLM recruiting to the ubiquitin-interacting motifs of RAP80, which is vital to maintain genome stability and suppressing tumourigenesis (Tikoo et al., 2013)
RFP	K27	inhibiting apoptosis and promoting cell survival and proliferation	tumor suppression	PTEN	E3 ubiquitin ligase RFP interacts with PTEN through K27-linked ubiquitination diminishing the effect of AKT signaling, involving in tumor suppression regulation (Lee et al., 2013)
FAAP20	K63	DNA damage repair and genome maintenance	leading to hematologic defects and cancer in patients	FANCA	FAAP20 binds K-63–linked ubiquitin chains in a UBZ domain-dependent manner, modulating DNA damage repair and genome maintenance (Ali et al., 2012)
LUBAC	M1	DNA damage-induced apoptosis	enhancing sensitivity to chemodrug in cancer cells	NEMO	LUBAC-mediated NEMO linear ubiquitination promotes TAK1 and IKK activation, protecting cells from DNA damage-induced apoptosis (Niu et al., 2011)
TRAF4 (RNF83)	K63	DNA damage	overcome chemoresistance in colorectal cancer	CHK1	CHK1 K63-linked ubiquitination is mediated by TRAF4, which is essential for CHK1phosphorylation and activation during DNA damage response, and is close to cell proliferation, colony formation in colorectal cancer (Yu et al., 2020b)
LUBAC	M1	regulating cell activation and death	promoting breast cancer	NEMO	Epsins 1 and 2 interact with LUBAC, promoting NEMO linear ubiquitination and resulting in breast cancer development (Song et al., 2021)
DZIP3	K63	regulating cell cycle	promoting cancer cell growth, migration, and invasion	Cyclin D1	DZIP3/hRUL138 stabilizes and ubiquitinated Cyclin D1 protein through K63-linked ubiquitination, and closely related with cell cycle progression, cancer cell growth, invasion, migration (Kolapalli et al., 2021)
RNF138	K63	driving NF-kB activation and innate immunity	promoting NF-kB activation in lymphomas	MYD88L265P	RNF138 triggers K63-linked polyubiquitination of MYD88L265P, resulting in constitutive activation of NF-κB and the associated lymphomagenesis (Yu et al., 2020a)
RNF181	K63	endocrine resistance	facilitating breast cancer progression	ERα protein	RNF181 functions as E3 enzymes through K63-linked ubiquitination, which facilitates breast cancer progression (Zhu et al., 2020)
TRIM11	mono-ubiquitination	regulating estrogen-dependent gene expression	promoting cell growth and migration	Erα	TRIM11 interacts with the N terminal of ERα and maintains ERα stability through mono-ubiquitination, thus promoting cell growth and proliferation in breast cancer (Tang et al., 2020)
HUWE1	K27-, K29-	regulating DNA damage response	promoting radio-resistance of prostate cancer cells	JMJD1A	HUWE1 mediates the K27-/K29-linked ubiquitination of JMJD1A, enhancing c-Myc activity, promoting DSB repair and sensitizing the response of prostate cancer (Fan et al., 2020)
RNF6	K63	maintaining nuclear receptors	promoting cell proliferation	glucocorticoid receptor (GR)	RNF6 stabilize GR genes and enhances its transcriptional activity by catalyzing its K63-linked polyubiquitination, promoting MM cell proliferation and survival (Ren et al., 2020)
TRIM27		suppressing cell senescence	cell cycle dysregulation, tumor cell proliferation and migration	p21	TRIM27 regulates cell apoptosis, cell senescence through mediating the ubiquitination of p21 in breast cancer (Xing et al., 2020)
SPOP	K27	increasing DNA replication stress	sensitizing cancer cells to ATR inhibition	Geminin	SPOP binding Geminin catalyzes K27-linked poly-ubiquitination of Geminin, preventing DNA replication over-firing and sensitizing cancer cells to ATR inhibition (Ma et al., 2021)
NF-X1	K33	regulating glycine metabolism	preventing glioma tumor growth	GLDC	Acetylation of GLDC inhibits its enzymes activity, and facilitates K33-linked ubiquitination by NF-X1, regulating glycine metabolism and tumorgenesis (Liu et al., 2021)
HUWE1	K63	promoting c-Myc activity	promoting retinoblastoma cell proliferation	c-Myc	HELZ2 triggers K63-linked ubiquitination activity of c-Myc by HUWE1 to mediate retinoblastoma tumorgenesis (Dai et al., 2021)
RNF8	K63	activating AKT pathway	promoting lung cancer cell proliferation and resistance to chemotherapy	Akt	RNF8 mediates K63-linked ubiquitination of Akt, promoting lung cancer cell proliferation and resistance to DNA damage (Xu et al., 2021)
TRIM31	K63	stabilizing and activating p53	inhibiting breast cancer progression	p53	TRIM31 directly ubiquitinates p53 with K63-linked ubiquitination through its RING domain, activating p53 pathway, suppressing breast cancer progression (Guo et al., 2021)
TRIM15	K63	activating NF-κB and Akt signaling pathway	regulating cancer cell growth, proliferation, migration	ERK1/2	TRIM15 mediates K63-linked polyubiquitination of ERK1/2, then activating ERK signaling, leading to cell proliferation, migration and differentiation (Zhu et al., 2021)
NEDD4	K63	regulating cell-cell adhesion, mechanosensing and autophagy	involving in angiogenesis and tumor growth	IGPR-1	NEDD4 and UbcH6 are involved in the K63-linked ubiquitination of IGPR-1, regulating different cellular activities (Sun et al., 2021)
TRIM25	K63	activating AKT/mTOR signaling	promoting NSCLC cell survival and tumor growth	PTEN	TRIM25 directly interacts with PTEN and catalyzes its K63-linked ubiquitination, modulating PTEN signaling and involving in cell survival and tumor growth in NSCLC (He et al., 2022)
TRIM41	K63	innate antiviral response	NF-κB associated cancer	BCL10	TRIM41 modifies K63-linked polyubiquitination of BCL10, activating NF-κB and TBK1 signaling pathway (Yu et al., 2021)
HECTD3	K63	regulating inflammation	NF-κB associated cancer	TRAF3	HECTD3 interacts with TRAF3 via K63-linked polyubiquitination, reducing inflammation and faciliating NF-κB inflammation pathway (Zhou et al., 2021)
DZIP3	K63	driving cell cycle	promoting cancer progression	Cyclin D1	DZIP3 stabilizes Cyclin D1 by promoting K63-linked ubiquitination of Cyclin D1, driving cell cycle and cancer progression (Kolapalli et al., 2021)
TRIM22	K63	activating NF-κB signaling	promoting glioblastoma tumor growth	IKKγ	TRIM22 promotes K63-linked ubiquitination of IKKγ, leading to degradation of IκBα and NF-κB activation (Ji et al., 2021)

#### 2.2.3 Linear Ubiquitination in Tumorigenesis

M1-linked ubiquitination, specifically N-terminal Met1-linked ubiquitination, is able to form eight different inter-ubiquitin linkages via its N-terminal methionine (M1). It can be specifically catalyzed by LUBAC. For instance, EGFR recruits PKP2 to the plasma membrane and cooperates with LUBAC (HOIP), activating linear ubiquitination of NEMO, which is critical for tumor cell proliferation (Hua et al., 2021). Also, Epsins 1/2 promotes NEMO linear ubiquitination via LUBAC, driving breast cancer development (Song et al., 2021). Moreover, LUBAC (SHARPIN) regulated *β*-catenin activity through linear ubiquitination, promoting gastric tumorigenesis (Zhang et al., 2021). Interestingly, OTULIN exclusively cleaves M1-linked ubiquitination and exhibits a high affinity for linear ubiquitination. LUBAC and linear ubiquitination have been found to be relevant to TNF signaling. Interestingly, OTULIN was shown to remove linear ubiquitination from LUBAC-modified proteins, which is critical for various cellular activities (Draber et al., 2015). Moreover, the deubiquitinating enzyme, CYLD, was also identified for disassembly Met1-linked-Ub (mostly the immune system). A previous report demonstrated that modification of proliferating cell nuclear antigen (PCNA) induces apoptosis and inhibits tumor growth through the linear ubiquitin chain (Qin et al., 2018).

### 2.3 Lys63 Linkage

#### 2.3.1 The Writer Enzymes for the Lys63 Linkage

It has been well established that Lys63 chains (ubiquitin chains topology lysine 63 poly-ubiquitin linkages) regulate and trigger distinct cellular signaling, including kinase activation, signal transduction, protein trafficking, endocytosis and DNA repair (Spence et al., 1995; Hofmann and Pickart, 1999; Komander and Rape, 2012; Wu and Karin, 2015; Hrdinka et al., 2016). The E2 conjugating enzyme complex Ubc13/Uev1A preferentially assembles the K63-pUb chain (Smith et al., 2013; Zhang et al., 2018). Furthermore, Tax can be recruited to K63-pUb by E3 ligase RNF8 and Ubc13/Uev1A, which allows the activation of TGFβ-activating kinase 1 (TAK1), followed by multiple downstream signaling pathways such as the IKK and JNK pathways. These ultimately lead to DNA damage repair, cytokinesis, and the genomic instability in ATL cells (Ho et al., 2015; Lee et al., 2017). Similarly, tumor necrosis factor receptor associated factor 6 (TRAF6) can interact with the E2 conjunction enzyme Ubc13/Uev1A in a RING-dependent manner, catalyzing Lys63-linked TRAF6 auto-ubiquitination. This activates IKK and NF-κB, thereby promoting TAK1 and IKK to trigger spontaneous osteoclast differentiation (Lamothe et al., 2007a; Lenoir et al., 2021). Another report also showed that TRAF6, in a RING-dependent fashion, catalyzed auto-ubiquitination by conjugating with ubc13/Uev1A, activating the AKT pathway, and promoting cell migration in breast cancer (Lamothe et al., 2007b; Niu et al., 2021).

The Ubc13/Uev1A complex has been shown to conjugate Lys63-linked poly-ubiquitination of substrate proteins, which contribute to breast cancer metastasis via NF-кB signaling regulation (Wu Z. et al., 2014; 2017). It is also reported that Ubc13 can catalyze K63-linked protein poly-ubiquitination, which is indispensable for the activation of non-SMAD signaling by TAK1 and p38, whose activity controls breast cancer metastatic spread and lung colonization (Wu X. et al., 2014). Interacting with Ubc13, RNF213 mediates autoubiquitination and controls inflammatory responses and angiogenic activities (Habu and Harada, 2021). RNF8 was demonstrated to activate Ubc13 and recruit K63-linked poly-ubiquitin conjugation to histones H2A/H2AX, thus contributing to breast cancer predisposition (Vuorela et al., 2011). Moreover, RNF8 utilizes the RING domain, mediating Lys63-linked Ub chains, which is required for DNA double-strand break (DSB) signaling and the downstream BRCA1 tumor suppressor recruitment or lung cancer cell proliferation (Hodge et al., 2016; Xu et al., 2021). Inhibiting the Ubc13/Uev1A complex specifically, which is critical for Lys63-linked ubiquitination, promotes ubiquitin conjugation at the Lys147 site, thereby upregulating NF-кB signaling in multiple myeloma and other cancers (Gallo et al., 2014). UbcH6 and NEDD4 regulate angiogenesis and tumor growth (Sun et al., 2021). Ube2w accompanies the E3 ligase RNF4 function in distinct DNA repair pathways through Lys63-linked chains and BRIC6 (also named BRUCE) acting on K63-linked ubiquitinylate in unstimulated cells, which regulates the DNA double-strand break response through bridging USP8 and BRCT-repeat inhibitor of hTERT expression (BRIT1) in a deubiquitination manner (Ge et al., 2015; Maure et al., 2016). Poly-ubiquitination of histone H1 depends on Ubc13 and RNF8, which prolong pre-existing ubiquitin modifications to K63-linked chains, thereby stimulating RNF8-Ubc13 mediated DNA damage response (Mandemaker et al., 2017). SKP2 triggers non-proteolytic K63-linked ubiquitination, which is crucial for cancer initiation and progression by positively regulating cancer cell survival and glycolysis. The depletion of SKP2 restricts cancer stem cell proliferation and survival (Chan et al., 2013). However, the non-proteolytic K63-linked ubiquitination triggered by SKP2 can be reversed and modulated by debiquitinase ovarian tumor domain-containing protein 1 (OTUD1) (Yao et al., 2018) and USP10 (Liao et al., 2019). USP10 has been identified as a novel deubiquitinase of SKP2 that modulates and stabilizes SKP2. Indeed, USP10 can recognize and remove Lys63-linked ubiquitin chains from Bcr-Abl, leading to positive activities in chronic myeloid leukemia cells. OTUD1 interacts with p53 and is essential for constant stabilization of p53. Its overexpression dramatically induces the cell cycle and apoptosis (Mevissen et al., 2013; Piao et al., 2017). In addition, several more DUBs have been defined as having linkage specificity for Lys63-linked ubiquitination. CYLD, a tumor suppressor, inhibits NF-κB signaling by cleaving K63-linked ubiquitination of NEMO/IKKγ, thus reducing its stability and averting the IKK complex from phosphorylation of IκB (Chen T. et al., 2017). Furthermore, CYLD was associated with the catalytic LUBAC subunit HOIP to counteract Lys63-Ub and Met1-Ub conjugation to receptor-interacting protein kinase 2 (RIPK2), leading to restriction of innate immune signaling and cytokine production (Hrdinka et al., 2016). TRAF-binding protein domain (TRABID) was demonstrated to bind and cleave Lys63-linked ubiquitin moieties on APC tumor suppressor substrates, which led to the disruption of APC and activation of Wnt signaling in colorectal cancer cell lines (Tran et al., 2008). The USP17/TRAF2/TRAF3 complex acts to stabilize its client proteins, enhancing inflammatory responses and stemness in lung cancer cells (Lu et al., 2018). USP20 deubiquitinates TRAF6 and Tax, and may function as a key regulator of adult T cell leukemia (ATL) leukemogenesis through suppressing NF-κB activation (Yasunaga et al., 2011). In terms of actively removing Lys63-linked poly-ubiquitin chains on GβL, OTUD7B appears to be the primary regulator in governing mTOR2 integrity, which is essential for NF-κB activation in cancer biology (Wang B. et al., 2017). Further detailed analysis of Lys63-linked ubiquitination is required to better understand how ubiquitin chains function in numerous cellular events. However, identification of this linkage will provide an ideal point to understand the potential mechanism in the cellular regulation of the incoming ubiquitin research (Table 1, 2).

#### 2.3.2 Disassembly of Lys63 Poly-Ubiquitin Chains by DUBs

Although a number of DUBs have been identified, the disassembly ability of the substrate and its physiological role, especially for Lys63 poly-ubiquitin chains, is poorly defined. Previous studies have shown that K63-specific DUBs, A20, and CYLD present the anti-tumor effects by regulating NF-κB signaling (Heyninck and Beyaert, 2005; Sun, 2010; Meng et al., 2021; Zhu et al., 2021). USP10 reduces K63-linked ubiquitination chains and functions as a tumor suppressor (He et al., 2021). Stimulated by the pro-inflammatory cytokine TNF, A20 exerts two major functions: sequential de-ubiquitination and catalyzing receptor-interacting protein (RIP), resulting in downregulation of NF-κB signaling. Absence of A20 led to hyper-accumulation of Lys63 poly-ubiquitin in NF-κB signaling. Like A20, CYLD disassembles the Lys63 poly-ubiquitin recruiting signal and degrades IKKκ. In this way, USP4 and OTUD1 cleaves K63-linked ubiquitination chains, and regulates downstream NF-*κ*B signaling (Fan et al., 2011; Liang et al., 2013; Wu et al., 2022) (Table 3).

#### 2.3.3 The Multifaceted Functions of Lys63 Poly-Ubiquitin Chains in Cancer

Lys63-linked chains have been described as non-degradative signals and are the core of inflammatory system and the NF-κB pathway, which is involved in either mono-ubiquitination or poly-ubiquitination. TGFβ type I receptor (TBRI) was shown to interact with TRAF6 through Lys63-dependent poly-ubiquitination in order to promote tumor invasion (Mu et al., 2011). It has also been reported that polo-like kinase 1 (PLK1) phosphorylates kruppel-like factor 4 (KLF4), leading to the recruitment of TRAF6, resulting in Lys63-linked ubiquitination and promoting nasopharyngeal tumorigenesis (Mai et al., 2019). Moreover, TRAF6 functions in hepatocarcinogenesis by regulating TRAF6/HDAC3/c-Myc signal axis. Mechanistically, TRAF6 interacts with histone deacetylase 3 (HDAC3) via Lys63-linked ubiquitin chains, which enhances c-Myc stability, and its overexpression has been proposed to facilitate HCC progression (Wu et al., 2020). Similarly, HUWE1 promotes K63-linked ubiquitinatination of c-Myc, thereby promoting retinoblastoma cell proliferation (Dai et al., 2021). It was also demonstrated that ASK1-dependent phosphorylation and lys63-linked poly-ubiquitination of LKB1 is critical for its activation. Tankyrase and RNF146 act as upstream regulators of the LKB1/AMPK pathway to promote Lys63-linked ubiquitination resulting in AMPK activation and tumor suppression (Li et al., 2019). Lys63-linked non-proteolytic ubiquitination of Hexokinase 2 by HectH9 effectively disrupts glycolysis activation and facilitates apoptosis in prostate cancer cells (Lee H. J. et al., 2019). SET domain bifurcated histone lysine methyltransferase 1 (SETDB1) methylates Akt and functions as a scaffold to recruit JMJD2A, which then binds TRAF6 and Skp2-SCF to the Akt complex, thereby promoting tumor development in lung cancer (Wang et al., 2019). Notably, numbers of E3 ligase have been discovered to catalyze K63-linked ubiquitination chains, which are involved in activating NF-κB or Akt signaling pathway, acting as tumor promoters and facilitating tumor progression (Ji et al., 2021; Xu et al., 2021; Yu et al., 2021; Zhou et al., 2021; Zhu et al., 2021; He et al., 2022). Lys63 poly-ubiquitin chains were also found to be involved in driving the cell cycle and promoting cancer progression (Kolapalli et al., 2021). In contrast, tripartite motif-containing 31 (TRIM31) directly ubiquitinates p53 via K63-linked ubiquitination, resulting in tumor-suppressing effects (Guo et al., 2021) (Table 2).

### 2.4 Lys6 Linkages–Tumor Suppressors

Lys6-linked ubiquitin chains are less abundant in resting cells and their functional implications are unclear (Durcan et al., 2014; Michel et al., 2017). It has been reported that BRCA1 can be autoubiquitinated and then bound by UBX domain containing protein 1 (UBXN1) through K6-linked poly-ubiquitin chains. Interestingly, UBXN1 regulates BRCA1 expression upon ubiquitination. Autoubiquitinated forms of BRCA1 act as tumor suppressors and inhibit their enzymatic function (Wu-Baer et al., 2010). Moreover, BRCA1 auto-ubiquitination occurs in a way that the BRCA1/BARD1 complex conducts polymerization by conjugation with K6-linked polymers, which imparts cellular properties to its natural enzymatic substrates (Wu-Baer et al., 2003), further linking BRCA1 auto-ubiquitination to the tumor suppressor. C terminus HSC70-interacting protein (CHIP) was reported to bind to fas-associated protein with death domain (FADD) to induce K6-linked poly-ubiquitination of FADD, which was demonstrated to be essential for the prevention of cell death (Seo et al., 2018).

It has also been revealed that community-based learning collaborative (CBLC) assembles K6- and K11-linked poly-ubiquitin on EGFR and positively regulates its stability. The sustained activation of EGFR is largely dependent on CBLC-mediated ubiquitination, and its dysregulation is preferentially destined for membrane recycling, which plays an important role in non-small-cell lung carcinoma (NSCLC) progression (Hong et al., 2018).

Structural findings indicated that USP30 efficiently cleaves Lys6-linked Ub chains, which abrogates parkin-mediated Ub-chain formation in mitochondria. Dysfunction of this novel distal Ub-recognition mechanism is associated with physiological disorders such as hepatocellular carcinoma (Sato et al., 2017; Wang et al., 2022). Although an extraordinary progress has been made over the last 2 decades, the detailed functional consequences of Lys6 modifications require further investigation (Tables 2, 3).

### 2.5 Lys27 linkages—emerging Tumor Promoter

Emerging investigations have demonstrated that K27-linked poly-ubiquitination is crucial for promoting cancer development, such as facilitating cell proliferation, invasion, and metastasis (Peng et al., 2011; Yin et al., 2019). ITCH, an E3 ubiquitin ligase, was shown to generate Lys27-linked poly-ubiquitination of the transcription factor TIEG1, which inhibits TIEG1 nuclear translocation and subsequent Treg development (Peng et al., 2011). Additionally, TGF-β, in the presence of cytokine IL-6, can efficiently promote mono- and poly-ubiquitination of TIEG1 and modulate Treg/Th17 differentiation (Peng et al., 2011). Yet, it seems that tumor immunity in TIEG1^−/−^ mice were apparently enhanced by hampering Treg development and increasing Th17 response, suggesting its pro-tumor effects (Peng et al., 2011). In the presence of proinflammatory cytokines, ITCH can catalyze BRAF to disrupt 14–3–3-mediated inhibition of BRAF kinase activity, resulting in ΜEK/ERK signaling activation. This ubiquitin function plays an essential role in supporting the proliferation and invasion abilities of melanoma cells (Yin et al., 2019). Another report connected Lys27-linked ubiquitination to melanoma cell invasive properties, in which HECT domain and ankyrin repeat containing E3 ubiquitin protein ligase 1 (HACE1) decorated fibronection with Lys27 Ub moieties (El-Hachem et al., 2018). As mentioned in this study, upregulation of fibronectin in turn modulates the transcription levels of integrin subunit alpha V (ITGAV) and integrin β1 (ITGB1), leading to an increased invasive power of melanoma cells (El-Hachem et al., 2018). TRAF4 was found to increase TrkA kinase activity through K27- and K29-linked ubiquitination upon nerve growth factor (NGF) stimulation, followed by the recruitment of downstream adaptor proteins and increased metastasis in prostate cancer (Singh et al., 2018).

Strikingly, reports have also revealed that Lys27-linked ubiquitination is implicated in the DNA damage response (DDR) and innate immunity. A previous study demonstrated that RNF168 can target the N-terminal tail of histones H2A/H2A.X, generating Lys27-linked ubiquitin chains. In response to DNA damage, K27 ubiquitination is the major source of PTMs that mark chromatin. Meanwhile, the activition of DDR can be inhibited by mutation of K27, which hinders the localization of 53BP1 and BRCA1 to DDR foci (Gatti et al., 2015). DDR is responsible for the recognition, signal transduction and repair of DNA damage. The inactivation of DDR will result in the accumulation of cell mutations and the increase of genomic instability, which play an essential role in cancer initiation (Klinakis et al., 2020). It is also indicated that K27 and the related DDR mediators may be the potential targets for the development of anti-tumor drugs. cGAS is subjected to K27 poly-ubiquitination by RNF185, facilitating cGAS-mediated innate immune response. TRIM-mediated ubiquitination by binding to residue K27 activates TBK1 recruitment to mitochondrial antiviral signaling (MAVS) and promotes innate immunity (Wang Q. et al., 2017). TRIM31 and TRIM40 can also mediate K27-linked ubiquitination, thereby regulating innate and adaptive immunity (Wang X. et al., 2021; Shen et al., 2021). Specifically, TRIM40 interacts with Riok3, resulting in RIG-I and MDA5 degradation through K27-linked ubiquitination, and negatively regulates innate immunity (Shen et al., 2021). Nevertheless, TRIM31 catalyzes K27-linked ubiquitination of SYK, facilitating antifungal immunity (Wang X. et al., 2021). Additionally, auto-ubiquitination of TRIM23 through K27-linked ubiquitination was found to mediate autophagy via activation of TBK1 (Sparrer et al., 2017). Moreover, E3 ubiquitin ligase Hectd3 decorates Stata3 with non-degradative K27-linked poly-ubiquitin chains and Malt1 with K27/K29-linked poly-ubiquitin chains, leading to signaling-related ubiquitination in neuroinflammation (Cho et al., 2019) (Table 3). In fact, the innate immune system plays a key role in the formation of tumor. The response of it is generally affected by a variety of immune cells and cytokines in tumor microenvironment (Wenbo and Wang, 2017). In this way, it is beneficial to clarify the regulatory effects of K27 on tumor innate immunity for understanding the mechanisms of cancer.

### 2.6 Lys29 and Lys33 linkage—possible Tumor Promoters

Recent proteomic data have identified the role of Lys29-and Lys33-linked ubiquitin chains in various biological processes, including the control of AMPK-mediated mitochondrial function and Wnt-induced transcription signaling (Jiang et al., 2015; Nusse and Clevers, 2017). AMPK-related kinases, AMPK-related kinase 5 (ARK5, also known as NUAK1) and microtubule-affinity-regulating kinase 4 (MARK4), which are mediated by unconventional Lys29/Lys33 linkage, are involved in cell polarity and proliferation (Al-Hakim et al., 2008). One of the underlying mechanisms is that USP9X specifically identifies Lys29/Lys33-conjugated ubiquitin chains on NUAK1 and MARK4 (Al-Hakim et al., 2008). However, it should be noted that the ubiquitination of NUAK1 and MARK4 suppresses their phosphorylations rather than restores their stability and facilitates LKB1 activation. Interestingly, the TRABID core domain N-terminal Npl4-like zinc finger (NAZF1) preferentially hydrolyzes K29/K33-linked diUb, and this novel AnkUBD displays TRABID linkage specificity (Licchesi et al., 2011). Additionally, TRABID interacts with APC tumor suppressor protein, recruits TCF target genes, and activates their transcription in colorectal cancer cells (Tran et al., 2008). The OUT family DUB TRABID preferentially abolishes Smad ubiquitination regulatory factor 1-induced K29/K33-linked poly-ubiquitin chains from UV radiation resistance associated gene (UVRAG), thereby promoting autophagosome maturation and inhibiting cell proliferation in hepatocellular cancer (Feng et al., 2019). Considering its linkage specificity, it would be insightful to explore the potential positive regulation of TRABID in cancer tumorigenesis via these two pathways.

K29-linked ubiquitin chains play important roles in driving cancer invasion and metastasis, as well as in the positive regulation of immunity (Singh et al., 2018; Cho et al., 2019; Gao et al., 2021). Several studies have also manifested that, with the assistance of Cbl-b and ITCH, T cell receptor-zeta (TCR-zeta) was decorated with a K33 linage, accompanied by positively activated T cells and immune responses (Huang et al., 2010). In addition, OTUD1 directly deubiquitinates the inhibitor SMAD7 of TGF-β pathway and aborates Lys33-linked poly-ubiquitin chains, which inhibits cell stemness and suppresses metastasis (Zhang et al., 2017). Acetylation of GLDC inhibits its enzymatic activity and facilitates K33-linked ubiquitination by NF-X1, thereby suppressing glioma tumor growth (Liu et al., 2021). Ultimately, Lys29-and Lys33-linked ubiquitin chains appear to have more complicated cellular functions that remain to be characterized (Table 2, 3).

### 2.7 Mixed Linkage and Branched Poly-Ubiquitination

Mixed linkage chains send mixed signaling messages that can be identified by different linkage-specific receptors. Our understanding of the mixed linkages and branched poly-ubiquitin is limited. Previous reports have elucidated that Tax in combination with UbcH2, Uhc5c, or UbcH7, can catalyze the construction of free mixed-linkage poly-ubiquitin chains, which are responsible for IKK-NF-κB activation and induction of T cell transformation (Wang et al., 2016). Furthermore, Brcc 36 isopeptidase complex (BRISC), the JAMM/MPN + family of DUBs, preferentially cleaves K63 linkages within mixed-linkage chains. In addition, RING1B was found to generate atypical mixed poly-ubiquitin chains and mediate mono-ubiquitination of H2A (Ben-Saadon et al., 2006). A recent study showed that SPOP triggered mixed-linkage ubiquitination of MyD88 in human lymphoma cells and mouse HSCs, suggesting that the SPOP-MyD88 pathway plays a critical role in hematopoietic neoplasms (Jin et al., 2020).

## 3 Conclusion and Furture Perspectives

Non-proteolytic ubiquitination, the molecular switch in cell fate regulation, plays a crucial role in post-translational protein modifications. Diverse ubiquitination enzymes are essential for ubiquitination linkages that are necessary for normal metabolism and physiological functions. Meanwhile, it is also the root cause of physiological disorders, such as cancer. The aberrant regulation of the UPS is typically achieved by ubiquitination enzymes, DUBs, 20S proteasome catalytic core particles and 19S proteasome regulatory particles (Rape, 2018). In general, specific ubiquitination enzymes determine ubiquitin linking with one of the seven lysine residues to form distinctive styles of poly-ubiquitin chains, deciding the fate of substrate proteins. It is noteworthy that the ubiquitin proteasome system functions as a theoretical target for drug screening, and the study of ubiquitination will provide more insights into the development of anti-tumor drugs. Recently, the quantities of E3s inhibitors have already been processed in preclinical models of cancer immunotherapy. It has been shown that E3 enzyme Smac mimetics (SMs) were the promising immune modulators for cancer therapy as the antagonists targeting E3 ligases IAPs (Cossu et al., 2019). The small molecule inhibitor AMG-232, targeting another E3-ubiquitin ligase oncogenic mouse double minute 2 homolog (MDM2), was shown to strengthen T cell killing of cancer cells, especially when combined with an anti-PD-1 monoclonal antibody (Sahin et al., 2020). Several other proteasome inhibitors have been confirmed to be clinically effective against malignancies as well. The neddylation (NAE) inhibitor pevonedistat has already been tested in multiple clinical trials and has shown positive effects in patients with AML or advanced solid tumors (Barghout and Schimmer, 2021). Small molecule inhibitors based on deubiquitinase have been widely used in experimental anti-tumor therapy, most of which are still in the preclinical research stage. Due to dose-limiting toxicity, the first deubiquitinase inhibitor VLX1570 was terminated in the clinical trial phase. No other deubiquitinase inhibitors have been approved for clinical studies since then. Also, the discovered deubiquitinase inhibitors related to tumor treatment are mainly concentrated in the USP family, and the relationship between the inhibitors of non-USP family members and the treatment of malignant tumors needs to be further studied (Zhang et al., 2022). Interestingly, the innovative approaches of proteolysis targeting chimeras (PROTACs) and molecular glues might facilitate clinical cancer therapy (Cruz Walma et al., 2022; Kung and Weber, 2022; Zhou and Xu, 2022). Consequently, it is essential further to investigate the role of ubiquitination enzymes in tumorigenesis. Meanwhile, targeting different ubiquitination, including K6-, K27-, K29-, K33-, K63-, and M1-linked poly-ubiquitination, may also be one of the directions for cancer drug discovery. Although great progresses have been achieved with the development of anti-cancer drugs aimed at the UPS, numerous challenges still stand in the way. Some candidate inhibitors have emerged as drug resistant or have limited efficacy in patients. Despite these questions, the discovery of new drugs targeting single or multiple segments of the UPS is still worthy of future research.
